# Socioeconomic inequalities in health-related functioning among people with type 2 Diabetes: longitudinal analyses in the Maastricht Study

**DOI:** 10.1186/s12889-023-17553-z

**Published:** 2024-01-03

**Authors:** Rachelle Meisters, Jeroen Albers, Bengisu Sezer, Bastiaan E. de Galan, Simone J. P. M. Eussen, Coen D. A. Stehouwer, Miranda T. Schram, Marleen M. J. van Greevenbroek, Anke Wesselius, Annemarie Koster, Hans Bosma

**Affiliations:** 1 Department of Social Medicine, Care and Public Health Research Institute (CAPHRI), Maastricht University, Duboisdomein 30, Maastricht, 6229 GT the Netherlands; 2 Care and Public Health Research Institute (CAPHRI), Maastricht University, Maastricht, the Netherlands; 3 Department of Internal Medicine, Maastricht University Medical Center+, Maastricht, the Netherlands; 4 CARIM School for Cardiovascular Diseases, Maastricht University, Maastricht, the Netherlands; 5 Department of Internal Medicine, Radboud University Medical Center, Nijmegen, the Netherlands; 6 Department of Epidemiology, Maastricht University, Maastricht, the Netherlands; 7 MHeNS School for Mental Health and Neuroscience, Maastricht University, Maastricht, the Netherlands; 8 Heart and Vascular Center, Maastricht University Medical Center (MUMC+), Maastricht, the Netherlands; 9 School of Nutrition and Translational Research in Metabolism (NUTRIM), Maastricht University, Maastricht, the Netherlands

**Keywords:** Health-related functioning, Patient-reported outcome measures, Socioeconomic inequalities, Type 2 Diabetes mellitus

## Abstract

**Background:**

Type 2 diabetes mellitus (T2DM) is a common chronic disease that disproportionally affects disadvantaged groups. People with a low socioeconomic position (SEP) have increased risk of T2DM and people with a low SEP and T2DM have higher HbA_1c_-levels compared to people with T2DM and high SEP. The aim of this study is to analyze longitudinal socioeconomic differences in health-related functioning in people with T2DM.

**Methods:**

Longitudinal data from 1,537 participants of The Maastricht Study with T2DM were used (32.6% female, mean (SD) age 62.9 (7.7) years). SEP was determined by baseline measures of education, occupation and income. Health-related functioning (physical, mental and social) was measured with the Short-Form Health Survey and the Impact on Participation and Autonomy survey (all scored from 0 to 100). Associations of SEP and health-related functioning were studied annually over a 10-year period (median (IQR) 7.0 (5.0) years, baseline 2010–2018) using linear mixed methods adjusting for demographics, HbA_1c_-levels and lifestyle factors.

**Results:**

Participants with a low SEP had significantly worse health-related functioning compared to those with a high SEP. For example, participants with low income had lower scores for physical (-4.49[CI -5.77;-3.21]), mental (-2.61[-3.78,-1.44]) and social functioning (-9.76[-12.30;-7.23]) compared to participants with high income on a scale from 0 to 100. In addition, participants with a low education significantly declined more over time in mental (score for interaction education with time − 0.23[-0.37;-0.09]) and social functioning (-0.44[-0.77;-0.11]) compared to participants with high education. Participants with low and intermediate incomes significantly declined more over time in physical functioning (-0.17 [-0.34, -0.01 and − 0.18 [-0.36, 0.00]) compared to participants with high income.

**Conclusions:**

Among people with T2DM, those with a lower SEP had worse health-related functioning in general than people with a higher SEP. Additionally, people with T2DM and low education developed poorer mental and social functioning over time compared to people with T2DM and high education. People with T2DM and low or intermediate income declined more in physical functioning over time than those with high incomes. In addition to HbA_1c_-levels and lifestyle patterns, more attention is needed for socioeconomic differences in health-related functioning for people living with T2DM.

**Supplementary Information:**

The online version contains supplementary material available at 10.1186/s12889-023-17553-z.

## Introduction

Type 2 diabetes mellitus (T2DM) is a common chronic disease in all populations, that disproportionally affects people with a low socioeconomic position (SEP) [1]. Socioeconomic inequalities have been systematically demonstrated in T2DM incidence [2], higher HbA_1c_-levels [3–5], quality of T2DM care [3] and T2DM complications [3, 6]. Across the board of these T2DM outcomes, systematic literature reviews show a socioeconomic gradient with people with a low SEP facing increased risks of T2DM compared to people with higher SEP. Furthermore, people with low SEP and T2DM have higher HbA_1c_-levels, possibly also due to difficulties in adhering to healthy lifestyles. Less is known about socioeconomic health differences and longitudinal changes in health-related functioning in individuals with T2DM.

In the conceptual framework by Brown et al. [1], SEP is related to various health outcomes through a number of pathways. Aside from the physiological health outcomes mentioned above, the authors also present health outcomes that cover a broader sense of health and health-related functioning. These broader health outcomes are measured by patient-reported outcome measures (PROMs). PROMS are increasingly recognized as important measures in realizing diabetic patient-centered care and clinical decision-making [7–9]. PROMS enable researchers and clinicians to understand the disease course and interventions in a more subjective, holistic and patient-centered perspective. A well-known PROM is the Short Form-36 Health Survey [10], also known as the SF-36. It assesses health-related functioning in both the physical and mental health domain. Another PROM has been developed specifically for assessing the burden of chronic diseases on social functioning, the Impact on Participation and Autonomy (IPA) questionnaire [11]. The IPA questionnaire has been used to study social participation (such as social relationships, autonomy in self-care, mobility, leisure and family role) in diverse patients groups. For example, in patients from a rehabilitation facility of an academic hospital in the Netherlands [11], in people with chronic disease in the Netherlands [12], in people with depression, T2DM or COPD in the Netherlands [13] in patients with multiple sclerosis, rheumatoid arthritis or spinal injury in the UK [14] and people with T2DM in Iran [15]. In the Dutch T2DM study, lower SEP was associated with lower social participation for people with T2DM compared to people without T2DM [13]. In the Iranian T2DM study, lower levels of participation were found for people with uncontrolled T2DM (vs. controlled T2DM, as determined by internal specialists and endocrinologists), with higher age and with lower income and occupational status [15].

In examining health-related functioning for T2DM populations, the few studies that included socioeconomic factors used cross-sectional study designs [13, 15–17], limiting causal conclusions on the associations found between low SEP and poorer health-related functioning. The aim of this study is, therefore, to examine socioeconomic inequalities in health-related functioning over time among people with T2DM. This research has used data from a large subsample of people with T2DM, participating in The Maastricht Study, who were followed up to 10 years (median (IQR) 7.0 (5.0) years). Taking into account previous research, establishing socioeconomic gradients in HbA_1c_-levels [3–5] (a measure of disease control) and lifestyle factors in T2DM populations [18, 19], the current study will analyze longitudinal socioeconomic differences in health-related functioning independent of HbA_1c_-levels and lifestyle factors.

## Methods

We used data from The Maastricht Study, an observational prospective population-based cohort study. The rationale and methodology have been described previously [20]. In brief, the study focuses on the etiology, pathophysiology, complications and comorbidities of type 2 diabetes mellitus (T2DM) and is characterized by an extensive phenotyping approach. Eligible for participation were all individuals aged between 40 and 75 years and living in the southern part of the Netherlands. Participants were recruited through mass media campaigns and from the municipal registries and the regional Diabetes Patient Registry via mailings. Recruitment was stratified according to known T2DM status, with an oversampling of individuals with T2DM, for reasons of efficiency. The present report includes cross-sectional data from the first 7689 participants, who completed the baseline survey between November 2010 and December 2017. The examinations of each participant were performed within a time window of three months. The study has been approved by the institutional medical ethical committee (NL31329.068.10) and the Minister of Health, Welfare and Sports of the Netherlands (Permit 131088-105234-PG). All participants gave written informed consent. The participants were followed up to ten years after baseline (median (IQR) 7.0 (5.0) years). Annual follow-up data on health related functioning were available for 86.1% (year 1), 75.2% (year 2), 69.1% (year 3), 61.0% (year 4), 62.4% (year 5), 53.7% (year 6), 52.5% (year 7), 43.3% (year 8), 27.3% (year 9), and 10.6% (year 10) of these participants at time of analyses, follow up is ongoing. After excluding missing responses, 1,537 complete cases remained for education, 1,214 for income and 634 for occupational status groups.

### Measures

Prevalent T2DM was defined in accordance with WHO 2006 criteria [21]. All participants underwent a standardized oral glucose tolerance test after overnight fasting. Blood samples were collected at baseline and 120 min after the consumption of a 75 gram glucose drink. Participants who were insulin-dependent or with a fasting glucose level higher than 11.0 mmol/L (as determined by finger prick) did not undergo this test. Participants on diabetes medication and who were not previously diagnosed with type 1 diabetes were also considered to have T2DM.

### Health-related functioning

#### Physical and mental functioning

The SF-36 survey is a multidimensional scale for determining health status and health-related functioning [10]. The SF-36 contains eight themes: (1) physical functioning, (2) role limitations due to physical health problems, (3) bodily pain, (4) general health, (5) vitality, (6) social functioning, (7) role limitations due to emotional problems, and (8) mental health. The SF-36 responses are recalculated to a score range from 0 to 100, with higher scores indicating better functioning. This study focused on the physical and the mental component summary scores of the SF-36 survey [22], both of which were measured at baseline and annually during follow-up. The physical component summary score is calculated based on four themes; physical functioning, role limitations due to physical health problems, bodily pain and general health. The mental component summary score is based on the scores for the remaining four themes of vitality, social functioning, role limitations due to emotional problems and mental health.

### Social functioning

The Impact on Participation and Autonomy (IPA) questionnaire evaluates the perceived burden of a chronic disease on individual participation and autonomy [11]. Its four domains include (1) autonomy indoors, (2) family role, (3) autonomy outdoors and (4) social life and relationships. It has been developed and validated in the Netherlands [11] and applied in national [11–13] and international populations [14, 15]. This study focused on the autonomy outdoors subscale of the questionnaire. This subscale includes 5 questions, for example, “your ability to visit friends, neighbors or acquaintances when you want to is …” or “your ability to spend your (spare) time the way you want to (what, when and how long) to is…”. All 5 questions of the IPA subscale are reported in Supplementary Table 1. The questions are answered on a five-point Likert scale. The responses are recalculated to a score range from 0 to 100, with higher scores indicating better social functioning.

### Socioeconomic position

This study used three indicators of socioeconomic position, namely highest completed level of education, standardized household income and occupation status. Participants were asked about the highest education level they had completed in categories. Education level was categorized as low (none, (un-) completed primary or lower vocational education), intermediate (intermediate vocational or higher general secondary education) and high (higher vocational or university education). Participants were also asked to indicate their net monthly household income in 19 categories (ranging from <€750,- until >€5000,-). The midpoint of the reported household income (set at €600,- for the lowest category and €6000,- for the highest category) was divided by the square root of the household size to achieve an equivalent value per person [23, 24]. The equivalent household income was categorized in low, intermediate and high income based on tertiles. In an open-ended question, participants were asked to describe their current occupation. The answers were coded in accordance with the International Standard Classification of Occupations 2008 (ISCO-08) categories by a trained coder [25]. The ISCO codes were converted to the International Socio-Economic Index of Occupational Status (ISEI-08) to indicate occupational class status on a continuous scale. The ISEI-08 was categorized as low, intermediate and high occupational status based on tertiles.

### Covariates

Covariates included age, sex, marital status, HbA_1c_-levels, smoking, diet (including alcohol consumption) and physical activity. Marital status is categorized as (1) single, (2) married, domestic partnership, civil union or living together, (3) widowed and (4) divorced or separated. HbA_1c_-levels were determined in venous blood samples collected after an overnight fast. Participants answered whether they (1) never smoked, (2) were former smokers or (3) are current smokers. Based on a food-frequency questionnaire [26], the Dutch Healthy Diet index (DHD) [27], a measure of diet quality, a sum score was calculated to indicate adherence to Dutch dietary guidelines. Finally, based on the CHAMPS questionnaire [28], self-reported moderate to vigorous physical activity was determined in hours per week for each participant.

### Statistical analyses

Baseline lifestyle characteristics, HbA_1c_-levels, and health-related functioning were compared across SEP groups in terms of education, income and occupational status (low, intermediate and high). To investigate the longitudinal relationships between SEP and health-related functioning, multilevel modeling for repeated measures were used. Models were built up in a step-wise manner. The first model was adjusted for demographic factors (age, sex and marital status). The second model was additionally adjusted for HbA_1c_-levels. The third model was additionally adjusted for lifestyle factors (smoking status, diet and physical activity). Analyses accounted for the interaction between SEP indicators and time in order to assess the impact of SEP on health-related functioning over time. For robustness of the results, both categorical and continuous measures of SEP and time were used and the linear mixed methods were checked with and without repeated measures. The interaction with SEP and sex were tested and the interactions between SEP and time were also checked for interactions of time with all other covariates. The significance level was set at alpha = 5%. All analyses were conducted in SPSS 28 [29].

## Results

### Descriptive statistics

Of the 1,537 participants with T2DM, 32.6% were female and the mean (SD) age at baseline was 62.9 (7.7) years (see Table 1). The majority of the participants was married, in a domestic partnership, civil union or living together. The median (IQR) standardized household income was €1679,- (€1049,-). Almost half of the participants had a low level of education and a low level of occupational status. The mean (SD) score for physical functioning was 47.1 (9.5), for mental functioning 53.5 (8.5) and for social functioning 79.7 (17.6). The number of missings per variable are reported in Supplementary Table 2.

**Table 1:** Descriptive statistics (n = 1,537)

		Total	Education			
**Variable**		**Mean (SD) / N (%)**	**Low** **(N = 680)**	**Intermediate (N = 424)**	**High** **(N = 433)**	**p-value**
**Age**		62.7 (7.8)	64.3 (7.2)	61.4 (8.2)	62.4 (7.7)	< 0.001
**Sex**	Female	413(33.6%)	269 (39.6%)	132 (31.1%)	100 (23.1%)	
	Male	1,036 (67.4%)	411 (60.4%)	292 (68.9%)	333 (76.9%)	< 0.001
**Marital status**	Single	112 (7.3%)	48 (7.1%)	30 (7.1%)	34 (7.9%)	
	Married, domestic partnership, civil union or living together	1,226 (79.7%)	526 (77.4%)	347 (81.8%)	364 (79.9%)	
	Widowed	87 (5.7%)	55 (8.1%)	21 (5.0%)	11 (2.5%)	
	Divorced, Separated	109 (7.1%)	51 (7.5%)	26 (6.1%)	32 (7.4%)	0.026
**Household income (median/IQR)**		€1679,- (€1049,-)	€1503,- (€685,-)	€1856,- (€840,-)	€2210,- (€1113,-)	< 0.001
**Highest completed education**			680 (44.2%)	424 (27.6%)	433 (28.2%)	
**Occupation status**	Low	278 (43.9%)	176 (69.3%)	88 (47.3%)	14 (7.3%)	
	Intermediate	198 (31.3%)	65 (25.6%)	61 (32.8%)	72 (37.3%)	
	High	157 (24.8%)	13 (5.1%)	37 (19.9%)	107 (55.4%)	< 0.001
**HbA**_1c_ -level (mmol/mol)		50.0 (11.3)	50.9 (10.9)	50.6 (11.4)	49.4 (11.8)	0.084
**Smoking**	Never	473 (30.8%)	193 (28.4%)	136 (32.1%)	144 (33.3%)	
	Former	856 (55.7%)	378 (55.6%)	237 (55.9%)	241 (55.7%)	
	Current	208 (13.5%)	109 (16.0%)	51 (12.0%)	48 (11.1%)	0.087
**Self-reported MVPA (hours/week)**		4.3 (4.2)	3.7 (4.0)	4.8 (4.4)	4.9 (4.2)	< 0.001
**Diet score, including alcohol intake (DHD)**		80.5 (14.8)	79.3 (14.8)	80.4 (14.7)	82.6 (14.7)	0.001
**Physical Component Score (SF-36)**		47.1 (9.5)	45.0 (10.1)	47.4 (9.0)	49.9 (7.9)	< 0.001
**Mental Component Score (SF-36)**		53.5 (8.5)	53.2 (8.5)	53.9 (8.2)	53.6 (8.8)	0.401
**Impact on Participation and Autonomy score (IPA)**		79.7 (17.6)	76.9 (18.7)	80.5 (16.5)	83.5 (16.0)	< 0.001

Low education: No education, (un)completed primary education, or lower vocational education. Intermediate education: intermediate vocational education or higher secondary education. High education: higher vocational education or university education. HbA_1c_-levels: level of glycated hemoglobin, average blood sugar level. MVPA: moderate to vigorous physical activity. DHD: Dutch Healthy Diet. SF-36: Short form health survey

Table 1 and Supplementary Table 3 show lifestyle characteristics, HbA_1c_-levels and health-related functioning at baseline, per SEP group. Participants with a low education spent less time on physical activity, and had poorer diet scores and higher HbA_1c_-levels than participants with higher education. Participants with a low education also reported poorer physical and social functioning than people with high education. Similar differences were found between participants with low and high incomes for physical activity, diet scores, smoking, HbA_1c_ -levels, and physical, mental and social functioning. For occupational status groups, similar significant differences were found in physical, mental and social functioning.

### Longitudinal associations

**Fig. 1:**
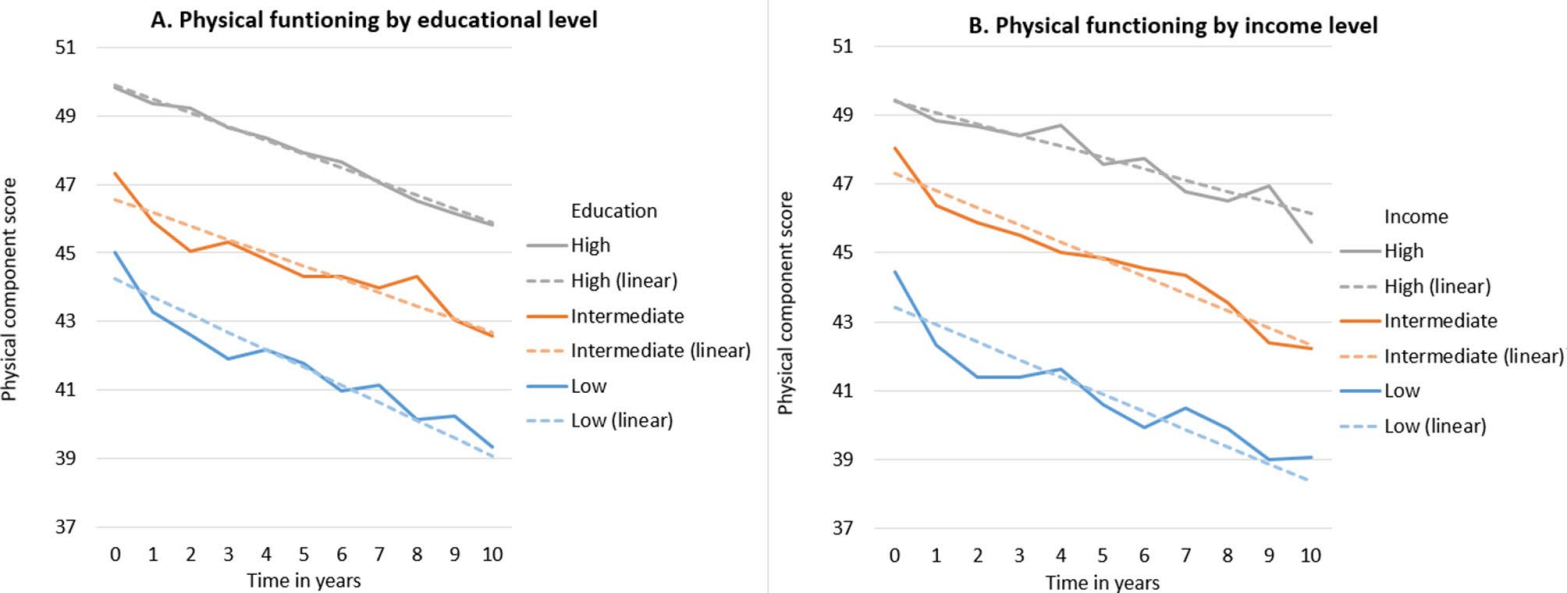
Estimated values for physical functioning, by time and SEP

**Fig. 2:**
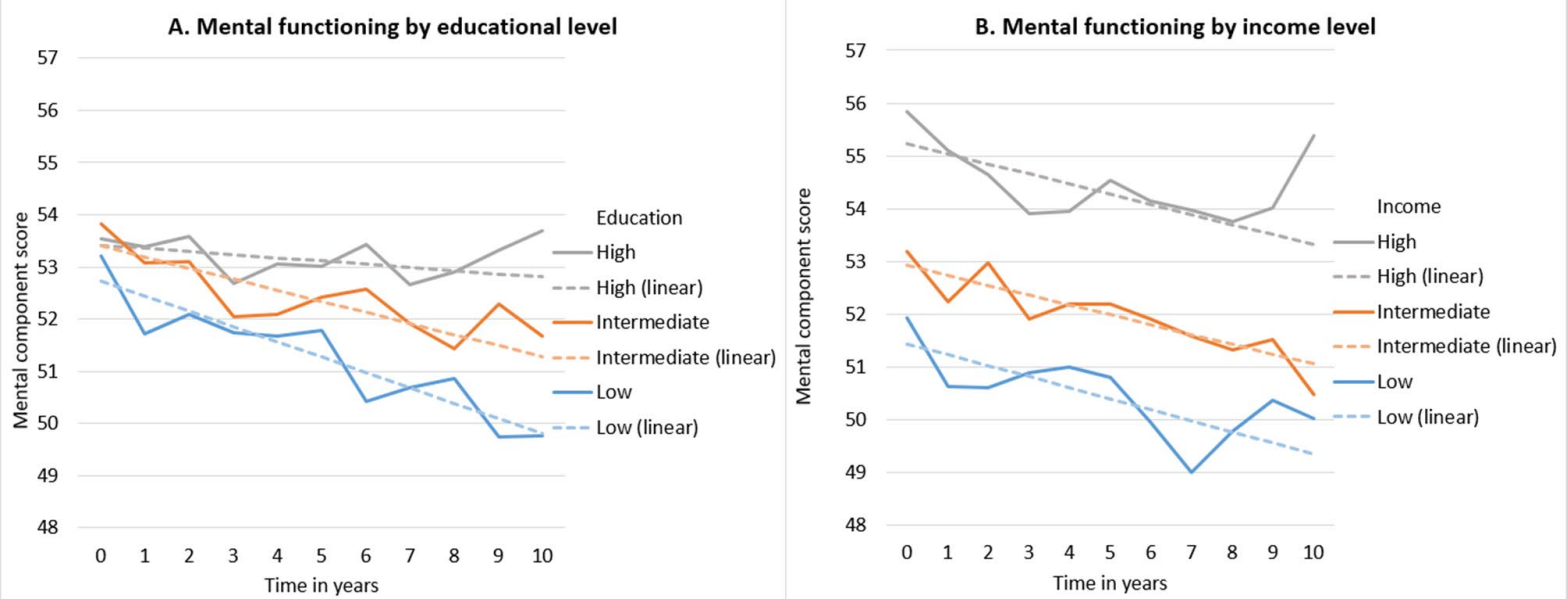
Estimated values for mental functioning, by time and SEP

Table 2 shows the socioeconomic differences in health-related functioning at baseline and over time, after adjustment for demographics, HbA_1c_-levels, and lifestyle variables. All models are presented in a stepwise manner in Supplementary Tables 4, 5, 6. The inclusion of demographic factors, HbA_1c_-levels and lifestyle factors did not substantially affect the influence of education, income and occupation on health-related functioning. Significant differences in physical and social functioning at baseline are reported for different educational groups. On a scale ranging from 0 to 100, participants with low (-3.88 [95% CI -4.98, -2.79]) and intermediate education had a lower score (-2.88 [-4.05, -1.71]) on baseline physical functioning than participants with high education. Similarly, participants with low and intermediate education had a lower score (-5.83 [-7.96, -3.70] and − 4.11 [-6.40, -1.83]) on baseline social functioning compared to participants with high education. In addition there were significant interaction effects of education and time on mental (P-value for interaction = 0.029) and social functioning (P-value for interaction = 0.031), see Table 2; Figs. 1A and 2 A. The interaction estimate for participants with low education was − 0.23 (-0.37, -0.09) for mental functioning and − 0.44 (-0.77, -0.11) for social functioning compared to participants with high education. In other words, participants with low education developed poorer mental and social functioning over time compared to people with high education.

**Table 2:** Socioeconomic differences in health-related functioning for people with T2DM at baseline and over time, adjusted for demographics, HbA1c-levels and lifestyle

	Estimate (95% CI)	Physical functioning	Mental functioning	Social functioning
Education (n = 1,537)	high	ref	ref	ref
	intermediate	**-2.88 (-4.04, -1.71)**	0.29 (-0.77, 1.35)	**-4.11 (-6.40, -1.83)**
	low	**-3.88 (-4.98, -2.79)**	-0.22 (-1.20, 0.77)	**-5.83 (-7.96, -3.70)**
	high education*time	Ref	Ref	Ref
	intermediate education*time	0.01 (-0.15, 0.17)	-0.15 (-0.30, 0.00)	-0.21 (-0.56, 0.15)
	low education*time	-0.12 (-0.27, 0.03)	**-0.23 (-0.37, -0.09)**	**-0.44 (-0.77, -0.11)**
Income (n = 1,214)	high	ref	ref	ref
	intermediate	**-1.66 (-2.80, -0.50)**	**-2.09 (-3.15, -1.03)**	**-5.72 (-8.01, -3.43)**
	low	**-4.49 (-5.77, -3.21)**	**-2.61 (-3.78, -1.44)**	**-9.76 (-12.30, -7.23)**
	high income *time	ref	ref	Ref
	intermediate income *time	**-0.17 (-0.34, -0.01)**	0.01 (-0.15, 0.16)	-0.15 (-0.51, 0.20)
	low income *time	**-0.18 (-0.36, 0.00)**	-0.02 (-0.19, 0.16)	-0.36 (-0.75, 0.03)
Occupation (n = 634)	high	ref	ref	ref
	intermediate	-1.70 (-3.50, 0.10)	-1.11 (-2.76, 0.54)	-1.28 (-4.88, 2.31)
	low	**-4.42 (-6.14, -2.71)**	**-1.81 (-3.38, -0.23)**	**-7.17 (-10.59, -3.75)**
	high occupation *time	ref	Ref	Ref
	intermediate occupation *time	0.03 (-0.21, 0.26)	0.13 (-0.11, 0.36)	-0.07 (-0.60, 0.46)
	low occupation *time	0.03 (-0.19, 0.25)	0.06 (-0.16, 0.28)	0.07 (-0.43, 0.57)

Significant findings in bold. The interaction estimate for participants with low education was for example, -0.23 (-0.37, -0.09) for mental functioning and -0.44 (-0.77, -0.11) for social functioning compared to participants with high education. In other words, participants with low education developed poorer mental and social functioning over time compared to people with high education.

Results adjusted for age, sex, marital status, HbA_1c_-levels, diet (incl. alcohol intake), physical activity and smoking. The dashed lines represent the results with time as a continuous variable and the solid lines represent the results with time as a categorical variable.

**Fig. 3:**
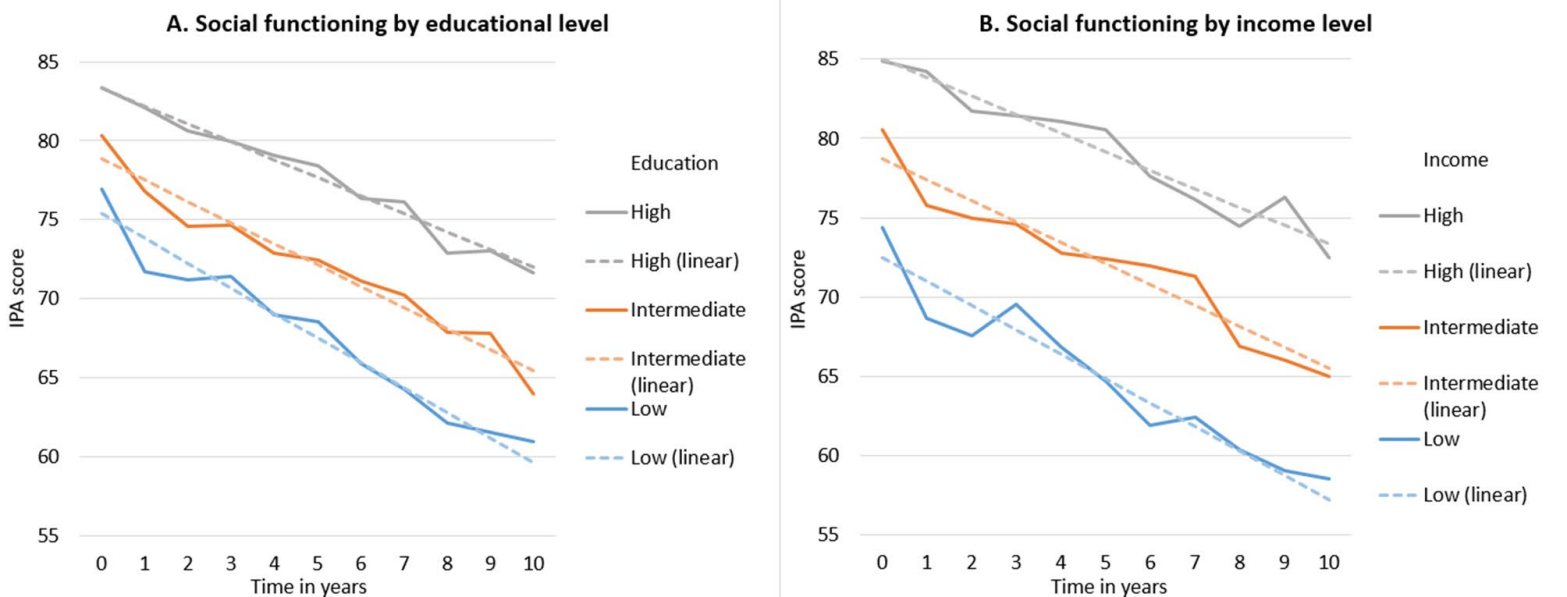
Estimated values for social functioning, by time and SEP

For income, significant differences were found at baseline for all three outcomes. Participants with low and intermediate incomes had a lower score (-4.49 [-5.77, -3.21]) and − 1.66 [-2.82, -0.50]) for physical functioning compared to participants with high incomes. Similarly, for mental functioning, participants with low and intermediate incomes had lower scores (-2.61 [-3.78, -1.44] and − 2.09 [-3.15, -1.03]) compared to participants with high incomes. Finally, participants with low and intermediate incomes also had lower scores (-9.76 [-12.30, -7.23] and − 5.72 [-8.01, -3.43]) for social functioning compared to participants with high incomes. Significant interaction effects were found for income and time on physical functioning (P-value for interaction = 0.045). The interaction estimate for participants with low income was − 0.18 (-0.36, 0.00) and for intermediate income − 0.17 (-0.34, -0.01) for physical functioning compared to participants with high incomes, see Table 2; Figs. 1B, 2B and 3B.

Results adjusted for age, sex, marital status, HbA_1c_-levels, diet (incl. alcohol intake), physical activity and smoking. The dashed lines represent the results with time as a continuous variable and the solid lines represent the results with time as a categorical variable.

For occupational status, significant differences were found at baseline for physical and social functioning, see Table 2; Fig. 3. Participants with low occupational status had a lower score (-4.42 [(-6.14, -2.71)] for physical functioning than participants with high occupational status. Furthermore, participants with low occupational status had a lower score for mental and social functioning (-1.81 [-3.38, -0.23]) and − 7.17 [-10.59, -3.75], respectively) than participants with high occupational status, see Table 2 and Supplementary Figures S1, S2 and S3.

Results adjusted for age, sex, marital status, HbA_1c_-levels, diet (incl. alcohol intake), physical activity and smoking. IPA: Impact on participation and autonomy. The dashed lines represent the results with time as a continuous variable and the solid lines represent the results with time as a categorical variable.

No statistically significant interactions with SEP and sex were found. To check the robustness of the results, the measure of time was also treated as a categorical variable. The results of these analyses showed similar trends to the main analyses (where time is treated as a continuous variable) and can be found in Figs. 1, 2 and 3. As an extra robustness check, analyses with time as categorical variable were run both with and without repeated measures. Both sets of the analyses produced nearly identical results, the results from analyses without repeated measures are represented with the solid lines in Figs. 1, 2 and 3. In addition, SEP measures were also included as continuous variables in sensitivity analyses. The results of these analyses (data not shown) showed similar trends to the main analyses for education and occupation. For income, the categorical variable had significant interactions with time for low and intermediate income on physical functioning, but the continuous variable did not. Finally, significant interactions effects between SEP and time were checked in models which included interaction effects of time with all other covariates. Of the three significant interactions between SEP and time on health-related functioning (education on mental, education on social and income on physical functioning), two interactions remained significant (education on mental, income on physical functioning) even when adjusting for interaction effects of time with all other covariates simultaneously. The interaction effect of low education and time on social functioning became insignificant when adjusting for the interaction effect of time and age, see Supplementary Table 7.

## Discussion

This study aimed to analyze longitudinal socioeconomic differences in health-related functioning among a large sample of people with T2DM over 10 years of follow-up (median (IQR) 7.0 (5.0) years). Health-related functioning was operationalized in terms of physical, mental and social functioning. This study shows general socioeconomic differences and widening inequalities over time in physical, mental and social functioning for people with T2DM. People with T2DM reported lower health-related functioning in general among those with lower education, income, and occupational groups than their counterparts in high education, income and occupational groups. In addition, this study shows that lower education predicts greater decline in mental functioning over time. The decline in physical functioning over time is also more substantial for people with low or intermediate incomes compared to people with high incomes. All of the studied effects of SEP on health-related functioning over time remained statistically significant with adjustments for demographic and lifestyle characteristics and HbA_1c_-levels. The results therefore indicate that people with T2DM and low SEP are faced with accumulation of disadvantages. Not only are people with low SEP at higher risk of T2DM [2], once they have T2DM, their level of glucose control is poorer and lifestyle patterns unhealthier, and they experience worse health-related functioning at similar HbA_1c_-levels and lifestyle patterns in general, and over time, they decline more in health-related functioning.

Previous cross-sectional research in T2DM populations established that low education is associated with poor physical functioning compared to higher levels of education [16]. In addition, a US neighborhood-based study found that people living in poorer neighborhoods reported poorer physical and mental functioning compared to people living in wealthier neighborhoods [17]. The current study extends these insights by showing poorer physical, mental and social functioning for lower educational, income and occupational groups with longitudinal, individual-based data among people with T2DM. The results show consistent disadvantageous patterns for people with intermediate or low SEP. General health-related functioning scores dropped between − 1.81 (-3.38, -0.23) and − 9.76 (-12.30-7.23) points when comparing people with high SEP to people with intermediate or low SEP. In addition, modest yearly decline was found for people with low or intermediate SEP, ranging between − 0.17 (-0.34, -0.01) and − 0.44 (-0.77, -0.11) lower scores per year. However modest, these numbers represent extra yearly decline in health-related functioning, on top off the already disadvantageous main effect of low and intermediate SEP on health-related functioning. In accordance with previous research, the current study also confirmed that people with low SEP are more at risk for high HbA_1c_-levels [3–5] and disadvantageous lifestyle factors [1, 18, 19]. The observed findings for socioeconomic differences in health-related functioning in general and inequalities over time are independent of HbA_1c_-levels and lifestyle factors.

The current study has several strengths related to the study design, the outcome variables and the included covariates. First, this study allows for longitudinal analyses as it followed respondents for a period of up to 10 years, with a median (IQR) follow-up period of 7.0 (5.0) years. Furthermore, the sample of roughly 1,500 people with T2DM (complete cases) allows for refined (sensitivity) analyses. Second, this study combined two constructs that allowed for a more diverse operationalization of health-related functioning. The results do not only consider the most commonly researched and validated patient-reported outcome measures, physical and mental functioning [30], but also social functioning. To our knowledge, this is the first study to analyze socioeconomic differences in social functioning for people with T2DM longitudinally. The results indicate that people with T2DM and low(er) SEP have worse social functioning than people with T2DM and high SEP. The social functioning scale questions participants on their ability to spend their leisure time and to interact with others as much as they want to. The results therefore suggest that people with T2DM and low SEP struggle more with social participation in society than people with T2DM and high SEP. Third, the current study builds on previous literature by including a set of covariates regarding lifestyle patterns and HbA_1c_-levels. This has enabled this study to demonstrate worsened health-related functioning for different socioeconomic groups over time, even when adjusted for HbA_1c_-levels and lifestyle factors. The results of this study demonstrate a multifaceted accumulation of disadvantages for people with T2DM and low SEP.

Some limitations of this study should be noted. First, the covariates in this study were taken at baseline, and changes during follow-up were not available for the analyses yet. Second, the sample of The Maastricht Study most likely still includes a healthier and higher SEP sample than the average T2DM population, as it is known that people with low SEP and poorer health are less likely to participate in research [31]. In addition, participants with poor health, low or intermediate SEP were more likely to drop out of the research during the study time compared to participants with good health and high SEP. Similar patterns were found for income, occupational status and for poor general health (as indicated in the prior survey). These patterns were also found for the share of participants not returning sent questionnaires. This implies that the findings from this study in the effects of SEP on health-related functioning may still be an underestimation of associations. In line with the healthy survivor bias, we cannot exclude the possibility that participants with low SEP are less likely to complete follow up measurements as they are known to have a shorter life expectancy. Third, the sample is based on a local population (the city of Maastricht and surroundings), and over 95% of the sample was Caucasian. This calls for more research in more ethnically diverse T2DM populations. Fourth, the sample at baseline was between 40 and 75 years old. As socioeconomic differences were already present at baseline measurement, this suggests that socioeconomic effects on health-related functioning in T2DM populations already take place before being included in this study. Perhaps even before reaching adulthood, as previous cross-sectional research suggests from a life-course perspective [32]. As measures of childhood SEP were not included in this study, future research may benefit from enriching longitudinal data with childhood SEP measures such as parental education, income and occupation status.

The results of this study provide directions for future research and implications for policies and clinical practices. This study shows an accumulation of disadvantages for people with low SEP and T2DM. This asks for a broader view on the cause of causes (of socioeconomic inequalities) in prevention policies [33]. The more traditional biomedical factors, such as lifestyle patterns and disease control in terms of HbA_1c_-levels, do not explain the observed socioeconomic inequalities in this study. Perhaps other disease characteristics than HbA_1c_-levels, or psychological processes that hinder coping with health-related functioning in people living with T2DM could help explain the observed socioeconomic inequalities. A previous study in a sample of people with depression, T2DM or COPD found that high levels of mastery helps coping and adapting to chronic conditions, resulting in better physical, mental and social functioning [13]. Or other societal, systemic factors that hinder inclusion or rehabilitation for people with T2DM and low SEP. A study analyzing to the work environment found low job control to be an important factor in explaining socioeconomic inequalities in T2DM [34]. Finally, overall quality of care has been systematically proven to be lower for people with lower SEP and T2DM [3]. More research is needed to unravel the possible underlying mechanisms between low SEP and poor health-related functioning. The insights of future research studying these mechanisms are necessary in developing appropriate policies and interventions for T2DM prevention and care. For clinical settings, this study provides relevant insights into the many disadvantages that people with low SEP face. They are not only at a higher risk to develop T2DM and have higher HbA_1c_-levels, but also report poorer quality of life in terms of physical, mental and social functioning, irrespective of HbA_1c_-levels and lifestyle factors. This accumulation of adversities should be recognized and targeted. The study also builds upon previous literature that not only biomedical factors should be monitored, but that monitoring PROM’s provides additional insights in T2DM disease course and quality of life for people with T2DM.

## Conclusion

In individuals with T2DM, socioeconomic health differences are apparent in health-related functioning. Even with similar demographics, HbA_1c_-levels and lifestyle patterns, people with T2DM and low SEP have worse physical, mental and social functioning at baseline compared to people with T2DM and high SEP. Moreover, differences in health-related functioning were more pronounced between different levels of education and income over time. Beyond HbA_1c_-levels and lifestyle patterns, more attention is needed for socioeconomic differences in health-related functioning for people living with T2DM, both in research and in clinical practice.

### Electronic supplementary material

Below is the link to the electronic supplementary material.

Supplementary Material 1: Tables and figures

## Data Availability

The datasets used and/or analysed during the current study are not publicly available but are available from the corresponding author on reasonable request.
