# Supplementary material for: Socioeconomic inequalities in health-related functioning among people with type 2 Diabetes: longitudinal analyses in the Maastricht Study
Source: BMC Public Health. 2024 Jan 3;24:73. doi: 10.1186/s12889-023-17553-z (PMC10763122; doi:10.1186/s12889-023-17553-z)
Supplement: Supplementary file 1 — Supplementary Material 1: Tables and figures [file 12889_2023_17553_MOESM1_ESM.docx]

**Supplementary Materials**

| **Supplementary Table 1. Questions for ‘Autonomy Outdoors’ subscale from the Impact on Participation and Autonomy survey [11]** |
| --- |
| 1. In general, your ability to visit friends, neighbors or acquaintances when you want to is… |
| 1. In general, your ability to make trips or go on a vacation when you want to is… |
| 1. In general, your ability to spend your (spare) time the way you want to (what, when and how long) is… |
| 1. In general, the frequency you visit friends, neighbors or acquaintances when you want to is… |
| 1. In general, your ability to live your life the way you want to is… |

The questions are answered on a five-point Likert scale, with ‘Very good’, ‘Good’, ‘Fair’, ‘Poor’, or ‘Bad’ as answer categories.

| **Supplementary Table 2. Missings (n=2,004)** | |
| --- | --- |
| **Variable** | **N (%)** |
| Age | 0 (0%) |
| Sex | 0 (0%) |
| Marital status | 26 (1.3%) |
| Household income | 606 (30.2%) |
| Highest completed education | 45 (2.2%) |
| Occupational status | 1174 (58.6%) |
| HbA1c-level | 3 (0.1%) |
| Smoking | 31 (1.5%) |
| Self-reported MVPA (hours/week) | 327 (16.3%) |
| Diet score, including alcohol intake (DHD) | 164 (8.2%) |
| Physical Component Score (SF-36) | 106 (5.3%) |
| Mental Component Score (SF-36) | 106 (5.3%) |
| Impact on Participation and Autonomy score (IPA) | 61 (3.0%) |

HbA_1c_-levels: level of glycated hemoglobin, average blood sugar level. MVPA: moderate to vigorous physical activity. DHD: Dutch Healthy Diet.

| **Supplementary Table 3. Lifestyle factors, HbA1c-level and health-related functioning per SEP group, at baseline** | | | | | | | | |
| --- | --- | --- | --- | --- | --- | --- | --- | --- |
|  | Smoker, current | Self-reported MVPA (hours/week) | Diet score, including alcohol intake (DHD) | HbA1c-level | Physical functioning | Mental functioning | Social functioning |  |
|  | N (%) | Mean (SD) | Mean (SD) | Mean (SD) | Mean (SD) | Mean (SD) | Mean (SD) |  |
| **Income** | 1,214 | 1,214 | 1,214 | 1,214 | 1,201 | 1,201 | 1,204 |  |
| High | 36 (9.2%) | 4.7 (4.2) | 80.9 (14.9) | 48.6 (9.5) | 49.5 (8.0) | 55.8 (6.9) | 85.0 (14.3) |  |
| Intermediate | 59 (12.9%) | 4.5 (4.0) | 81.8 (14.5) | 50.4 (11.4) | 48.0 (8.5) | 53.2 (8.0) | 80.7 (15.8) |  |
| Low | 69 (18.9%) | 3.6 (4.2) | 78.6 (14.7) | 52.3 (13.7) | 44.5 (10.8) | 51.9 (10.0) | 74.3 (21.2) |  |
| p-value | <0.001 | <0.001 | 0.006 | <0.001 | <0.001 | <0.001 | <0.001 |  |
| **Occupation** | 634 | 634 | 634 | 634 | 619 | 619 | 623 |  |
| High | 17 (10.8%) | 4.8 (4.2) | 82.5 (14.5) | 49.9 (9.8) | 50.5 (7.4) | 54.7 (6.8) | 84.0 (15.4) |  |
| Intermediate | 24 (12.1%) | 4.4 (4.0) | 81.3 (15.1) | 51.1 (12.9) | 47.7 (8.8) | 53.8 (9.0) | 81.4 (16.5) |  |
| Low | 39 (14.0%) | 4.0 (4.1) | 79.2 (13.5) | 52.1 (11.7) | 45.6 (10.3) | 52.3 (8.6) | 75.7 (18.9) |  |
| p-value | 0.811 | 0.121 | 0.059 | 0.172 | <0.001 | 0.012 | <0.001 |  |

HbA_1c_-levels: level of glycated hemoglobin, average blood sugar level. MVPA: moderate to vigorous physical activity. DHD: Dutch Healthy Diet.

| **Supplementary Table 4. Differences in educational groups and health-related functioning at baseline and over time (n=1,537), in unadjusted and adjusted models.** | | | | |
| --- | --- | --- | --- | --- |
|  | Estimate (95% CI) | Model 1 | Model 2 | Model 3 |
| Physical functioning | high education | ref | ref | ref |
|  | intermediate education | **-3.36 (-4.60, -2.12)** | **-3.18 (-4.40, -1.96)** | **-2.88 (-4.05, -1.71)** |
|  | low education | **-5.29 (-6.42, -4.15)** | **-4.99 (-6.11, -3.86)** | **-3.88 (-4.98, -2.79)** |
|  | high education*time | ref | ref | ref |
|  | intermediate education*time | 0.01 (-0.14, 0.17) | 0.01 (-0.15, 0.17) | 0.01 (-0.15, 0.17) |
|  | low education*time | -0.12 (-0.27, 0.03) | -0.12 (-0.26, 0.03) | -0.12 (-0.27, 0.03) |
| Mental functioning | high education | ref | ref | ref |
|  | intermediate education | 0.10 (-0.96, 1.17) | 0.17 (-0.89, 1.24) | 0.29 (-0.77, 1.35) |
|  | low education | -0.80 (-1.78, 0.18) | -0.69 (-1.67, 0.29) | -0.22 (-1.20, 0.77) |
|  | high education*time | ref | ref | ref |
|  | intermediate education*time | -0.15 (-0.30, 0.00) | -0.15 (-0.30, 0.00) | -0.15 (-0.30, 0.00) |
|  | low education*time | **-0.23 (-0.37, -0.09)** | **-0.23 (-0.37, -0.09)** | **-0.23 (-0.37, -0.09)** |
| Social participation | high education | ref | ref | ref |
|  | intermediate education | **-4.68 (-7.03, -2.34)** | **-4.44 (-6.78, -2.11)** | **-4.11 (-6.40, -1.83)** |
|  | low education | **-7.95 (-10.10, -5.79)** | **-7.54 (-9.69, -5.39)** | **-5.83 (-7.96, -3.70)** |
|  | high education*time | ref | ref | ref |
|  | intermediate education*time | -0.20 (-0.56, 0.15) | -0.20 (-0.56, 0.15) | -0.21 (-0.56, 0.15) |
|  | low education*time | **-0.44 (-0.77, -0.11)** | **-0.44 (-0.76, -0.11)** | **-0.44 (-0.77, -0.11)** |

Model 1: age, sex, marital status and time. Model 2: age, sex, marital status, time and HbA_1c_-levels. Model 3: age, sex, marital status, time, HbA_1c_-levels, diet (incl. alcohol intake), physical activity and smoking. No missings in independent variables, n=1,537. Significant estimates in bold (p<0.05). HbA_1c_-levels: level of glycated hemoglobin, average blood sugar level.

| **Supplementary Table 5. Differences in income groups and health-related functioning at baseline and over time (n=1,214), in unadjusted and adjusted models.** | | | | |
| --- | --- | --- | --- | --- |
|  | Estimate (95% CI) | Model 1 | Model 2 | Model 3 |
| Physical functioning | high income | ref | ref | ref |
|  | intermediate income | **-2.02 (-3.24, -0.81)** | **-1.78 (-2.99, -0.58)** | **-1.66 (-2.82, -0.50)** |
|  | low income | **-5.84 (-7.17, -4.52)** | **-5.34 (-6.66, -4.02)** | **-4.49 (-5.77, -3.21)** |
|  | high income *time | ref | ref | ref |
|  | intermediate income *time | **-0.17 (-0.34, -0.01)** | **-0.17 (-0.34, -0.01)** | **-0.17 (-0.34, -0.01)** |
|  | low income *time | **-0.18 (-0.36, 0.00)** | **-0.18 (-0.36, 0.00)** | **-0.18 (-0.36, 0.00)** |
| Mental functioning | high income | ref | ref | ref |
|  | intermediate income | **-2.25 (-3.32, -1.18)** | **-2.18 (-3.25, -1.11)** | **-2.09 (-3.15, -1.03)** |
|  | low income | **-3.22 (-4.38, -2.05)** | **-3.07 (-4.24, -1.89)** | **-2.61 (-3.78, -1.44)** |
|  | high income *time | ref | ref | ref |
|  | intermediate income *time | 0.01 (-0.15, 0.16) | 0.01 (-0.15, 0.16) | 0.01 (-0.15, 0.16) |
|  | low income *time | -0.02 (-0.19, 0.16) | -0.01 (-0.19, 0.16) | -0.02 (-0.19, 0.16) |
| Social participation | high income | ref | ref | ref |
|  | intermediate income | **-6.30 (-8.66, -3.94)** | **-6.02 (-8.38, -3.67)** | **-5.72 (-8.01, -3.43)** |
|  | low income | **-12.00 (-14.57, -9.42)** | **-11.41 (-14.00, -8.82)** | **-9.76 (-12.30, -7.23)** |
|  | high income *time | ref | ref | ref |
|  | intermediate income *time | -0.15 (-0.51, 0.20) | -0.15 (-0.51, 0.20) | -0.15 (-0.51, 0.20) |
|  | low income *time | -0.36 (-0.75, 0.03) | -0.35 (-0.75, 0.04) | -0.36 (-0.75, 0.03) |

Model 1: age, sex, marital status and time. Model 2: age, sex, marital status, time and HbA_1c_-levels. Model 3: age, sex, marital status, time, HbA_1c_-levels, diet (incl. alcohol intake), physical activity and smoking. No missings in independent variables, n=1,214. Significant estimates in bold (p<0.05). HbA_1c_-levels: level of glycated hemoglobin, average blood sugar level.

| **Supplementary Table 6. Differences in occupation groups and health-related functioning at baseline and over time (n=634), in unadjusted and adjusted models.** | | | | |
| --- | --- | --- | --- | --- |
|  | Estimate (95% CI) | Model 1 | Model 2 | Model 3 |
| Physical functioning | high occupation | ref | ref | ref |
|  | intermediate occupation | **-2.42 (-4.36, -0.48)** | -2.10 (-3.99,- 0.20) | -1.70 (-3.50, 0.10) |
|  | low occupation | **-5.76 (-7.58, -3.93)** | **-5.23 (-7.02, -3.43)** | **-4.42 (-6.14, -2.71)** |
|  | high occupation *time | ref | ref | ref |
|  | intermediate occupation *time | 0.03 (-0.21, 0.26) | 0.03 (-0.21, 0.26) | 0.03 (-0.21, 0.26) |
|  | low occupation *time | 0.03 (-0.19, 0.25) | 0.03 (-0.19, 0.25) | 0.03 (-0.19, 0.25) |
| Mental functioning | high occupation | ref | ref | ref |
|  | intermediate occupation | -1.37 (-3.04, 0.31) | -1.27 (-2.94, 0.41) | -1.11 (-2.76, 0.54) |
|  | low occupation | **-2.24 (-3.82, -0.67)** | **-2.08 (-3.67, -0.50)** | **-1.81 (-3.38, -0.23)** |
|  | high occupation *time | ref | ref | ref |
|  | intermediate occupation *time | 0.12 (-0.11, 0.36) | 0.12 (-0.11, 0.36) | 0.13 (-0.11, 0.36) |
|  | low occupation *time | 0.06 (-0.16, 0.28) | 0.06 (-0.16, 0.28) | 0.06 (-0.16, 0.28) |
| Social participation | high occupation | ref | ref | ref |
|  | intermediate occupation | -2.27 (-6.02, 1.47) | -1.84 (-5.55, 1.88) | -1.28 (-4.88, 2.31) |
|  | low occupation | **-9.02 (-12.54, -5.49)** | **-8.32 (-11.84, -4.81)** | **-7.17 (-10.59, -3.75)** |
|  | high occupation *time | ref | ref | ref |
|  | intermediate occupation *time | -0.07 (-0.60, 0.47) | -0.07 (-0.60, 0.47) | -0.07 (-0.60, 0.46) |
|  | low occupation *time | 0.07 (-0.43, 0.57) | 0.07 (-0.43, 0.57) | 0.07 (-0.43, 0.57) |

Model 1: age, sex,marital status and time. Model 2: age, sex, marital status, time, and HbA_1c_-levels. Model 3: age, sex, marital status, time, HbA_1c_-levels, diet (incl. alcohol intake), physical activity and smoking. No missings in independent variables, n=634. Significant estimates in bold (p<0.05). HbA_1c_-levels: level of glycated hemoglobin, average blood sugar level.

**Figure S1: Estimated values for physical functioning, by time and occupational status**

**
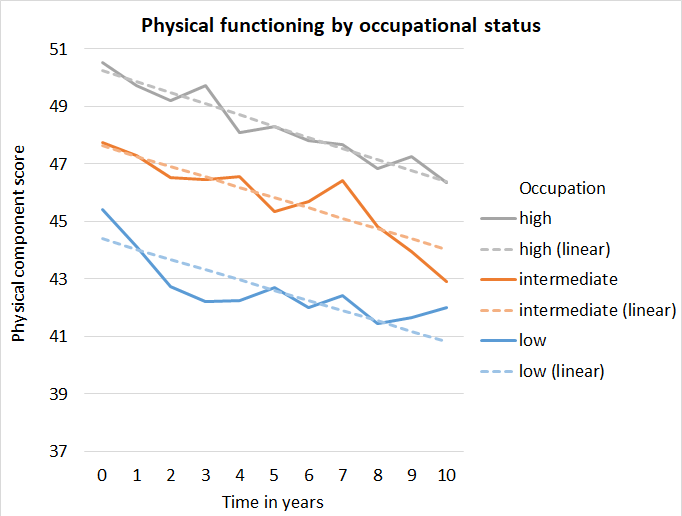
**

Results adjusted for age, sex, marital status, HbA_1c_-levels, diet score (including alcohol consumption), physical activity and smoking. The dashed lines represent the results with time as a continuous variable and the solid lines represent the results with time as a categorical variable.


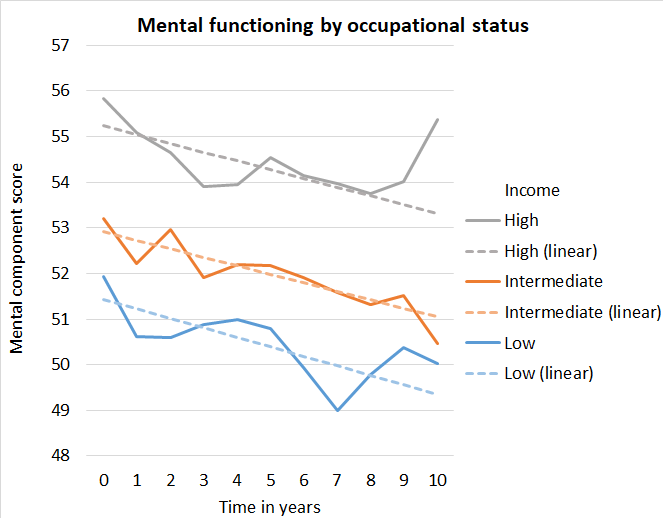
**Figure S2: Estimated values for mental functioning, by time and occupational status**

Results adjusted for age, sex, marital status, HbA_1c_-levels, diet score (including alcohol consumption), physical activity and smoking. The dashed lines represent the results with time as a continuous variable and the solid lines represent the results with time as a categorical variable.


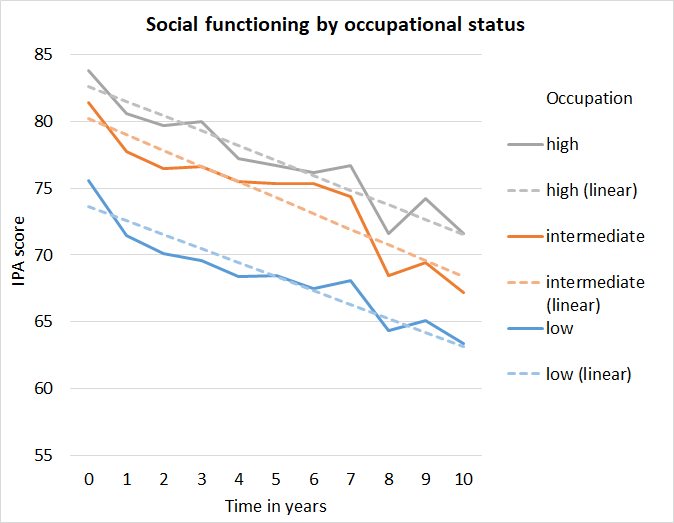
**Figure S3: Estimated values for social functioning, by time and occupational status**

Results adjusted for age, sex, marital status, HbA_1c_-levels, diet score (including alcohol consumption), physical activity and smoking. IPA: impact on participation and autonomy. The dashed lines represent the results with time as a continuous variable and the solid lines represent the results with time as a categorical variable.

| **Supplementary Table 7. Interaction effect of time*SEP on health-related functioning when adjusting for interaction effects of time and other covariates.** | | | | | |
| --- | --- | --- | --- | --- | --- |
| Education (N=1,537) | model 3 | model 3 +  age*time | model 3 +  sex*time | model 3 +  marital status*time | model 3 +  hba1c-level*time |
| Mental functioning |  |  |  |  |  |
| High education*time | ref | ref | ref | ref | ref |
| Intermediate education*time | -0.15 (-0.30, 0.00) | **-0.17 (-0.32, -0.02)** | **-0.16 (-0.31, -0.01)** | **-0.15 (-0.31, 0.00)** | -0.15 (-0.30, 0.00) |
| Low education*time | **-0.23 (-0.37, -0.09)** | **-0.20 (-0.34, -0.06)** | **-0.24 (-0.39, -0.10)** | **-0.23 (-0.38, -0.09)** | **-0.23 (-0.38, -0.09)** |
| Social functioning |  |  |  |  |  |
| High education*time | ref | ref | ref | ref | ref |
| Intermediate education*time | -0.21 (-0.56, 0.15) | -0.28 (-0.61, 0.06) | -0.21 (-0.57, 0.14) | -0.20 (-0.55, 0.15) | -0.20 (-0.55, 0.16) |
| Low education*time | **-0.44 (-0.77, -0.11)** | -0.27 (-0.59, 0.04) | **-0.45 (-0.78, -0.12)** | **-0.42, (-0.75, -0.09)** | **-0.42 (-0.75, -0.09)** |
| Income (N=1,214) |  |  |  |  |  |
| Physical functioning | |  |  |  |  |
| High income*time | ref | ref | ref | ref | ref |
| Intermediate income *time | **-0.17 (-0.34, -0.01)** | **-0.18 (-0.33, -0,02)** | **-0.18 (-0.34, -0.02)** | **-0.17 (-0.33, -0.01)** | **-0.17 (-0.33, -0.01)** |
| Low income *time | **-0.18 (-0.36, 0.00)** | **-0.20 (-0.37, -0.02)** | **-0.19 (-0.37, -0.01)** | **-0.18 (-0.36, -0.00)** | -0.18 (-0.36, 0.00) |

Model 3 adjusted for: age, sex, marital status, time, HbA_1c_-levels, diet score (including alcohol consumption), physical activity and smoking. Significant estimates in bold (p<0.05).

| **Supplementary Table 7. Continued** | | | | |
| --- | --- | --- | --- | --- |
| Education (N=1,537) | model 3 +  physical activity*time | model 3 + smoking*time | model 3 +  diet*time | model 3 +  all interactions |
| Mental functioning |  |  |  |  |
| High education*time | ref | ref | ref | ref |
| Intermediate education*time | -0.15 (-0.30, 0.00) | -0.15 (-0.30, 0.00) | -0.15 (-0.30, 0.00) | **-0.19 (-0.34, -0.04)** |
| Low education*time | **-0.22 (-0.36, -0.07)** | **-0.24 (-0.38, -0.09)** | **-0.23 (-0.37, -0.09)** | **-0.21 (-0.36, -0.06)** |
| Social functioning |  |  |  |  |
| High education*time | ref | ref | ref | ref |
| Intermediate education*time | -0.20 (-0.56, 0.15) | -0.20 (-0.56, 0.15) | -0.19 (-0.54, 0.16) | -0.24 (-0.58, 0.10) |
| Low education*time | **-0.38 (-0.71, -0.05)** | **-0.44 (-0.77, -0.11)** | **-0.41 (-0.74, -0.09)** | -0.11 (-0.44, 0.21) |
| Income (N=1,214) | | | | |
| Physical functioning | | | | |
| High income*time | ref | ref | ref | ref |
| Intermediate income*time | **-0.18 (-0.34, -0.01)** | **-0.17 (-0.34, -0.01)** | **-0.18 (-0.34, -0.02)** | **-0.18 (-0.34, -0.02)** |
| Low income*time | **-0.19 (-0.36, -0.01)** | **-0.19 (-0.37, -0.01)** | -0.18 (-0.35, 0.00) | **-0.19 (-0.37, 0.01)** |

Model 3 adjusted for: age, sex, marital status, time, HbA_1c_-levels, diet score (including alcohol consumption), physical activity and smoking. Significant estimates in bold (p<0.05).
